# Pre-eclampsia Complicated by HELLP Syndrome in a Primigravida: A Case Report

**DOI:** 10.7759/cureus.80770

**Published:** 2025-03-18

**Authors:** Nabeelah Ismail, Muhammad Faraaz Ismail, Eman Ibrahim

**Affiliations:** 1 General Medicine, Thumbay University Hospital, Ajman, ARE; 2 Obstetrics and Gynecology, Thumbay University Hospital, Ajman, ARE

**Keywords:** hellp syndrome, hypertensive disorders of pregnancy, intrauterine growth restriction, placental insufficiency, pre-eclampsia

## Abstract

Pre-eclampsia is a hypertensive disorder of pregnancy that can rapidly progress to HELLP syndrome, increasing maternal and fetal risks. We report a case of a 33-year-old primigravida at 28+1 weeks gestation who presented with severe hypertension, proteinuria, and reduced fetal movements. Despite initial management, she developed progressive thrombocytopenia, worsening liver function, and oligohydramnios, necessitating close monitoring. Biochemical markers indicated worsening disease, prompting an emergency cesarean section at 28+6 weeks, delivering a growth-restricted neonate. Postoperatively, the patient showed gradual improvement, and histopathology confirmed placental malperfusion. This case underscores the importance of early recognition, biochemical monitoring, and timely delivery in managing severe pre-eclampsia and preventing life-threatening complications.

## Introduction

Pre-eclampsia is a hypertensive disorder of pregnancy characterized by new-onset hypertension and end-organ dysfunction, typically occurring after 20 weeks of gestation. Affecting a significant number of pregnancies worldwide, it remains a leading cause of maternal and perinatal morbidity and mortality [1]. When complicated by HELLP syndrome, the risks to both mother and fetus increase substantially, often requiring urgent intervention to prevent adverse outcomes.

The pathophysiology of pre-eclampsia is multifactorial, involving abnormal placentation, endothelial dysfunction, and systemic inflammation [2]. Two major subtypes have been identified, early-onset (<34 weeks) and late-onset (>34 weeks) pre-eclampsia, with early-onset cases demonstrating more severe complications, including intrauterine growth restriction (IUGR) and placental insufficiency [3]. Despite its prevalence, early-onset pre-eclampsia with rapid progression to HELLP syndrome remains a critical clinical challenge, requiring vigilant monitoring and individualized management to optimize maternal-fetal outcomes.

A key feature of pre-eclampsia is placental ischemia and oxidative stress, which result in the release of anti-angiogenic factors leading to systemic endothelial dysfunction [3]. Moreover, HELLP syndrome represents a severe form of pre-eclampsia with increased maternal and fetal risks [4]. Delayed recognition of HELLP syndrome due to its atypical presentation can contribute to poor outcomes [4].

Several biochemical markers, including lactate dehydrogenase (LDH), aspartate aminotransferase (AST), alkaline phosphatase (ALP), and platelet count, are valuable in assessing disease severity. Studies indicate that elevated LDH levels correlate with increased maternal morbidity, hemolysis, and intensive care unit (ICU) admission rates [5].

This case report emphasizes the importance of recognizing rapidly progressing early-onset pre-eclampsia at 28 weeks and its transition to HELLP syndrome. By highlighting the evolving biochemical and clinical markers that guided timely intervention, this case contributes to the ongoing refinement of obstetric management strategies in such high-risk scenarios.

## Case presentation

Patient information and initial presentation

A 33-year-old primigravida was referred at 28+1 weeks gestation with severe hypertension (blood pressure of 170/110 mmHg), headache, dizziness, lethargy, and reduced fetal movements. Notable findings included 4+ proteinuria and a protein/creatinine ratio of 11.39 mg/mg (reference: less than 0.15 mg/mg). The patient had no known history of chronic hypertension. She also had no family history of chronic hypertension, pre-eclampsia, or other hypertensive disorders. However, she had a past history of controlled bronchial asthma, which necessitated cautious use of labetalol. On admission, laboratory tests revealed normal renal and liver function tests (LFTs) and a platelet count of 273 × 10³/µL (reference: 150 - 410 × 10³/µL). She was transferred to the ICU for close monitoring and management given the severity of hypertension and associated risks.

Clinical course and outcomes

Hospital Day 1

The patient was admitted to the ICU due to persistent severe hypertension (blood pressure of 170/110 mmHg) and the risk of complications. She was initiated on oral nifedipine 15 mg twice daily. Prior to transfer, she had received a loading dose of 4 g intravenous magnesium sulfate at the referring hospital as part of seizure prophylaxis. Upon admission, she received a second 12 mg dose of dexamethasone for fetal lung maturity, having received the first dose before transfer. Continuous fetal and maternal monitoring was established. An indwelling urinary catheter was placed to ensure accurate monitoring of her urine output. Repeat urinalysis continued to show heavy proteinuria (3+), and her platelet count was 273 × 10³/µL.

Hospital Day 2

**Figure 1:**
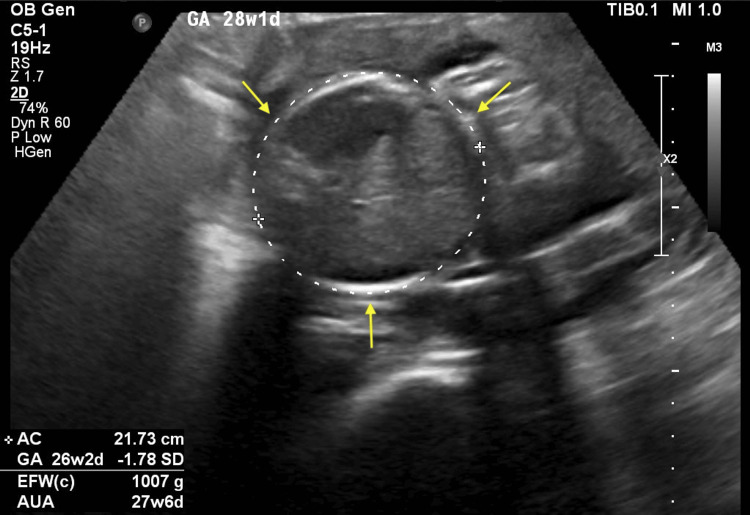
Obstetric ultrasound showing fetal abdominal circumference measuring 21.73 cm, indicated by white dotted lines and yellow arrows. The measurement corresponds to 26 weeks and two days of gestation, indicative of IUGR. AC, abdominal circumference; GA, gestational age; EFW, estimated fetal weight; AUA, average ultrasound age

**Figure 2:**
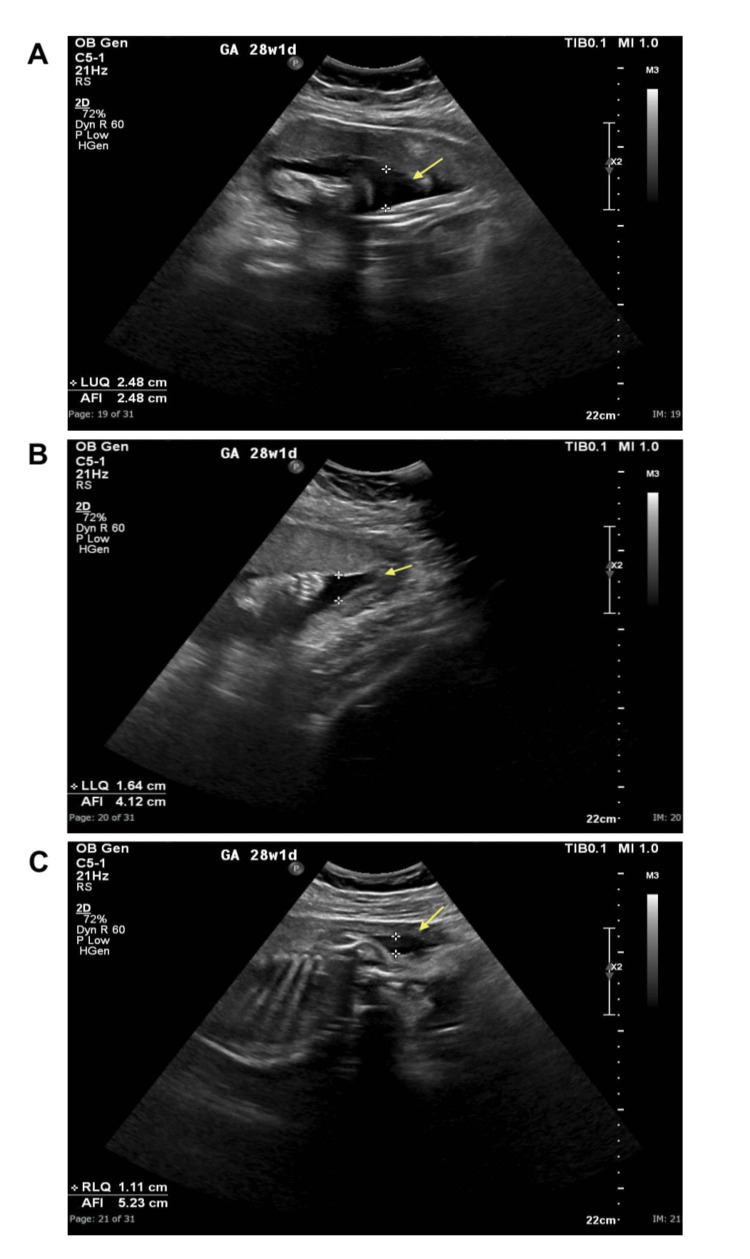
Obstetric ultrasound showing (A) the diameter of the LUQ fluid pocket measuring 2.48 cm, (B) the diameter of the LLQ fluid pocket measuring 1.64 cm, and (C) the diameter of the RLQ fluid pocket measuring 1.11 cm, with a total AFI of 5.2 cm, indicative of oligohydramnios. LUQ, left upper quadrant; LLQ, left lower quadrant; RLQ, right lower quadrant; AFI, amniotic fluid index

The patient remained hypertensive but stable. Blood pressure fluctuated between 154/95 mmHg and 170/110 mmHg, requiring an increase in nifedipine to 30 mg. Obstetric ultrasound showed a single live fetus with oligohydramnios and an amniotic fluid index (AFI) of 5.2 cm, as well as suspicion of IUGR due to reduced abdominal circumference measurements (as seen in Figure 1 and Figure 2), and an estimated fetal weight of 1007 g, which is below the 10th percentile (typically around 1110-1120 g), necessitating strict fetal surveillance. Laboratory investigations revealed a decreasing platelet count of 250 × 10³/µL but stable renal and liver function. Methyldopa was introduced to further manage hypertension.

Hospital Day 3

Despite antihypertensive therapy, blood pressure remained high (149/91 mmHg), and the patient developed symptoms of polydipsia and throat dryness. Laboratory findings included a protein/creatinine ratio of 7.52 mg/mg, mild hyperglycemia (GRBS (random blood sugar) 174 mg/dL), ALP 111 U/L (reference: 35 - 104 U/L), and AST 29 U/L (reference: up to 35 U/L). The platelet count dropped further to 178 × 10³/µL. Management included continued blood pressure control, fluid monitoring, and dietary adjustments. Enoxaparin (Clexane) 40 mg SC was introduced for thromboprophylaxis.

Hospital Day 4

The patient exhibited mild regurgitation after meals but had no neurological symptoms. Blood pressure readings fluctuated between 121/102 mmHg and 147/84 mmHg. A repeat ultrasound showed persistent oligohydramnios (AFI 6.1 cm) and fetal growth restriction. Close monitoring of fetal kick counts and serial CTGs was advised. Platelet count continued to decline to 145 × 10³/µL. LDH levels were 322 U/L (reference: up to 247 U/L), ALP 128 U/L, and AST 73 U/L, suggesting ongoing hemolysis.

Hospital Day 5

The patient developed worsening biochemical markers, with urinalysis showing protein 2+. The protein/creatinine ratio significantly increased to 25.55 mg/mg. She reported epigastric pain, raising concerns for HELLP syndrome. Given the increasing risk of eclampsia, she received another loading dose of 4 g intravenous magnesium sulfate, followed by a maintenance infusion of 24 g magnesium sulfate in 500 mL sodium chloride at a rate of 1 g/hour over 24 hours. A decision was made for an emergency cesarean section. Intraoperatively, labetalol 50 mg IV was administered for transient hypertension. The neonate, weighing approximately 1 kg, was transferred to the NICU. The placenta was sent for histopathological examination to assess for features consistent with pre-eclampsia and placental insufficiency.

Hospital Day 6 - (Postoperative Day 1)

The patient was monitored in the ICU postoperatively. She was hemodynamically stable with no signs of postpartum hemorrhage. Blood pressure was controlled with nifedipine and methyldopa. Laboratory results confirmed HELLP syndrome with a further decline in platelet count to 112 × 10³/µL. The Foley’s catheter was removed, and ambulation was encouraged.

Hospital Day 7 - (Postoperative Day 2)

The patient continued to recover with stable vital signs (blood pressure of 131/82 mmHg). Laboratory tests showed persistent thrombocytopenia (platelet count of 102 × 10³/µL ) with improving hepatic function. Urinalysis again showed protein 3+, and the protein/creatinine ratio improved to 3.34 mg/mg. ALP was 108 U/L, and AST 58 U/L, showing a trend toward resolution of liver dysfunction. Pain control was achieved with oral analgesics. Mobilization was encouraged, and oral intake was well tolerated.

Hospital Day 8 - (Discharge)

The patient was asymptomatic and hemodynamically stable (blood pressure of 129/87 mmHg). Her complete blood count showed an improving platelet count of 116 × 10³/µL. She was discharged on nifedipine 60 mg once daily, enoxaparin for 10 days, and instructed to monitor her blood pressure at home. Follow-up was scheduled within three days, with repeat complete blood count and LFTs in one week.

To facilitate interpretation, the patient's laboratory values and their trends have been organized into a table (Table 1), providing a clear overview of disease progression throughout hospitalization.

**Table 1:** Trends in proteinuria, creatinine, protein/creatinine ratio, platelet count, and liver function tests during hospitalization. ALP, alkaline phosphatase; AST, aspartate transferase; LDH, lactate dehydrogenase

Date	Proteinuria	Creatinine (reference: 0.51 - 0.95 mg/dL)	Protein/creatinine ratio (reference: less than 0.15 mg/mg)	Platelets (reference: 150 - 410 × 10³/µL)	ALP (reference: 35 - 104 U/L)	AST (reference: up to 35 U/L)	LDH (reference: up to 247 U/L)
Presentation	4+	0.65	11.39	273	-	26	198
Day 1	3+	-	-	273	-	-	-
Day 2	-	0.63	-	250	98	25	-
Day 3	-	-	7.52	178	111	29	-
Day 4	-	0.72	-	145	128	73	322
Day 5	2+	0.67	25.55	-	-	-	-
Day 6	-	-	-	112	101	56	-
Day 7	3+	0.53	3.34	102	108	58	-
Day 8	-	-	-	116	-	-	-

## Discussion

Pathophysiology, IUGR, and disease progression

Pre-eclampsia is characterized by abnormal placental development and vascular dysfunction, leading to widespread maternal endothelial damage [3]. Placental ischemia results in elevated oxidative stress, triggering the release of anti-angiogenic factors that impair maternal circulation [3]. In this case, progressively worsening platelet counts (from 273 × 10³/µL to 102 × 10³/µL) and elevated LDH (322 IU/L) suggested ongoing hemolysis and endothelial dysfunction, confirming the HELLP syndrome diagnosis [4].

Fetal ultrasound findings were consistent with IUGR, characterized by oligohydramnios (AFI 5.2-6.1 cm), decreased abdominal circumference measurements, and an estimated fetal weight below the 10th percentile. IUGR is a well-documented consequence of placental insufficiency in pre-eclampsia [3]. The presence of maternal vascular malperfusion in the histopathology confirmed the link between placental dysfunction and fetal growth restriction, necessitating urgent delivery [3]. 

The patient’s worsening protein/creatinine ratio (25.55 mg/mg) and urinalysis with persistent proteinuria indicated renal involvement, a hallmark of severe pre-eclampsia [2]. According to Sibai et al., renal dysfunction in pre-eclampsia is caused by glomerular endotheliosis, leading to impaired filtration and increased protein excretion [2].

In addition to fetal complications such as IUGR and oligohydramnios, severe pre-eclampsia and HELLP syndrome pose significant maternal risks, including eclampsia, pulmonary edema, acute kidney injury, hepatic rupture, and disseminated intravascular coagulation. These life-threatening complications necessitate intensive monitoring and timely intervention to prevent severe morbidity and mortality [2].

Biochemical markers and clinical correlation

LDH, a marker of cellular injury and hemolysis, was notably elevated in this patient. Research indicates that elevated LDH levels are associated with increased maternal complications and ICU admission rates [5]. This aligns with our case, where the patient required ICU admission and displayed hepatic impairment (AST: 73 U/L, ALP: 128 U/L) and progressive thrombocytopenia.

A retrospective study by Jayawardena et al. emphasized that early recognition and immediate delivery are crucial in managing HELLP syndrome [4]. In this case, the decision for emergency cesarean section was made based on worsening laboratory markers (platelets 102 × 10³/µL, AST 73 U/L) and maternal symptoms such as epigastric pain and uncontrolled hypertension, which are recognized indications for urgent delivery in severe pre-eclampsia [2].

Management and outcomes

The management of severe pre-eclampsia and HELLP syndrome is centered around blood pressure control, seizure prophylaxis, and timely delivery [1]. Nifedipine and methyldopa were used to maintain blood pressure, while magnesium sulfate prophylaxis was administered to reduce seizure risk [2]. 

The laboratory progression (LDH 322 U/L, AST 73 U/L, and a declining platelet count) highlights ongoing hemolysis and hepatic involvement, key features of HELLP syndrome [4]. Immediate delivery, guided by persistent maternal hypertension and epigastric pain, aligns with recommendations for severe pre-eclampsia management where deterioration is evident [1]. The histopathological findings of maternal vascular malperfusion corroborate the diagnosis of early-onset pre-eclampsia and support the decision for emergent cesarean delivery.

Similar to our case, a report by Almuhaytib et al. described a patient diagnosed with early-onset pre-eclampsia complicated by HELLP syndrome and IUGR, requiring urgent pregnancy termination due to severe maternal symptoms that did not improve with medical management [6]​. Their case highlights the importance of timely intervention in cases of severe pre-eclampsia, particularly when biochemical markers and clinical symptoms indicate maternal deterioration [6]. While their patient underwent medical termination at 25 weeks using misoprostol, our case required an emergency cesarean section at 28+6 weeks, reinforcing the individualized approach to management based on gestational age, fetal viability, and maternal status. This highlights the role of multidisciplinary decision-making in optimizing both maternal and neonatal outcomes in early-onset severe pre-eclampsia cases.

Given the significant maternal risks associated with HELLP syndrome, postpartum monitoring remains essential to prevent further complications. Postpartum complications remain a concern in HELLP syndrome, with risks including persistent hypertension, postpartum hemorrhage, delayed platelet recovery, and thromboembolic events. Close monitoring was emphasized to prevent these complications, and the patient was managed with continued antihypertensive therapy and thromboprophylaxis postoperatively [1,2].

Long-term neonatal outcomes

Preterm neonates born to mothers with HELLP syndrome are at an increased risk of long-term neurodevelopmental impairments, including cognitive deficits, motor dysfunction, and delayed language acquisition. The neonate in this case, delivered at 28+6 weeks with IUGR and oligohydramnios, faces an elevated risk of such complications due to the interplay of prematurity and placental insufficiency.

Early intervention programs have demonstrated efficacy in mitigating these risks. The Newborn Individualised Developmental Care and Assessment Program (NIDCAP) has been shown to significantly enhance neurobehavioral and neurological development in preterm infants by two weeks corrected age, emphasizing individualized, developmentally supportive NICU care [7]​. Additionally, a parent-guided early intervention program incorporating sensory stimulation and structured motor activities has been found to improve cognitive function at 18 months of corrected age, highlighting the role of parental involvement in optimizing neurodevelopmental outcomes [8].

Given these findings, implementing NIDCAP-based strategies during hospitalization and engaging parents in structured developmental interventions post-discharge could be critical in improving the long-term prognosis of neonates affected by HELLP syndrome [7,8]. 

## Conclusions

This case highlights the rapid progression of severe pre-eclampsia to HELLP syndrome, emphasizing the need for vigilant maternal and fetal monitoring to prevent life-threatening complications. The presence of symptoms suggestive of imminent eclampsia, such as headache, dizziness, and lethargy, necessitates urgent delivery to mitigate maternal and fetal risks. Early recognition of biochemical and clinical deterioration played a crucial role in guiding timely obstetric intervention, ultimately improving maternal and neonatal outcomes. The management of such cases requires a multidisciplinary approach, integrating close hemodynamic monitoring, biochemical surveillance, and individualized delivery planning. Given the high recurrence risk, future pregnancies for this patient will necessitate preconception counseling, early initiation of aspirin prophylaxis, and intensive maternal-fetal surveillance to optimize outcomes and reduce potential complications. Additionally, considering the neonate’s prematurity and IUGR, structured early intervention programs such as NIDCAP and parent-guided developmental care could play a pivotal role in improving long-term neurodevelopmental outcomes. Future research should focus on optimizing pre-eclampsia screening, prevention strategies, and the integration of neonatal interventions into standard care for high-risk infants.
